# A novel strategy of co-expressing CXCR5 and IL-7 enhances CAR-T cell effectiveness in osteosarcoma

**DOI:** 10.3389/fimmu.2024.1462076

**Published:** 2024-10-10

**Authors:** Xinhui Hui, Muhammad Asad Farooq, Yiran Chen, Iqra Ajmal, Yaojun Ren, Min Xue, Yuzhou Ji, Bingtan Du, Shijia Wu, Wenzheng Jiang

**Affiliations:** ^1^ Shanghai Key Laboratory of Regulatory Biology, School of Life Sciences, East China Normal University, Shanghai, China; ^2^ The First Dongguan Affiliated Hospital, Guangdong Provincial Key Laboratory of Medical Immunology and Molecular Diagnostics, Guangdong Medical University, Dongguan, China; ^3^ College of Life Science, Xinjiang Normal University, Urumqi, China

**Keywords:** CAR-T therapy, tumor microenvironment, T cell migration, osteosarcoma, CXCR5, IL-7

## Abstract

**Background:**

Solid tumors are characterized by a low blood supply, complex stromal architecture, and immunosuppressive milieu, which inhibit CAR-T cell entry and survival. CXCR5 has previously been employed to increase CAR-T cell infiltration into CXCL13+ cancers. On the other hand, IL-7 improves the survival and persistence of T cells inside a solid tumor milieu.

**Methods:**

We constructed a novel NKG2D-based CAR (C5/IL7-CAR) that co-expressed CXCR5 and IL-7. The human osteosarcoma cell lines U-2 OS, 143B, and Mg63 highly expressed MICA/B and CXCL13, thus presenting a perfect avenue for the present study.

**Results:**

Novel CAR-T cells are superior in their activation, degranulation, and cytokine release competence, hence lysing more target cells than conventional CAR. Furthermore, CXCR5 and IL-7 co-expression decreased the expression of PD-1, TIM-3, and TIGIT and increased Bcl-2 expression. Novel CAR-T cells show enhanced proliferation and differentiation towards the stem cell memory T cell phenotype. C5/IL7-CAR-T cells outperformed conventional CAR-T in eradicating osteosarcoma in mouse models and displayed better survival. Additionally, CXCR5 and IL-7 co-expression enhanced CAR-T cell numbers, cytokine release, and survival in implanted tumor tissues compared to conventional CAR-T cells. Mechanistically, C5/IL7-CAR-T cells displayed enhanced STAT5 signaling.

**Conclusion:**

These findings highlight the potential of CXCR5 and IL-7 co-expression to improve CAR-T cell therapy efficacy against osteosarcoma.

## 1 Introduction

Osteosarcoma (OS), while relatively uncommon in the general population, presents a notable surge in incidence during the pubertal growth spurt of young adults. It is, therefore, considered an orphan disease due to the inadequate population size affected by this disease compared to other forms of cancer (1, 2). Hypoxia and acidic microenvironment promote the release of pro-angiogenic factors (HIF and VEGF) by OS tumor cells to form highly vascularized tumors, which is thought to be the main reason for the high rate of metastasis and spread of OS. In addition, the extracellular matrix (ECM) composition of OS is significantly altered, and secretion is significantly increased, resulting in a harder matrix that makes it more difficult to infiltrate for immune cells (2). There is currently a marked stagnation in its treatment progress, while adjuvant chemotherapy has remained the primary therapeutic approach for the past four decades (1). Given the recent limitations of these treatments, innovating and implementing novel treatment options for OS is unequivocally important.

A diverse array of immunotherapies, including chimeric antigen receptor (CAR)-T cell therapy, are actively being employed to identify new treatment options for OS. The efficacy of CAR-T cell therapy in solid tumors still remains a challenge despite excellent remission rates in patients with hematological malignancies (3). For OS treatment, different targets for CAR-T cells are being explored to precisely mark and eradicate target-positive malignant cells (4, 5). One promising avenue is the development of CAR-T cells targeting the natural killer group 2D (NKG2D), which has exhibited significant efficacy in eradicating OS cells both *in vitro* and in murine models (6, 7). Some studies have shown that NKG2D-based CAR-T cells are quite safe for ligand-negative cells, including normal body tissues (6, 8, 9).

Solid tumors often create an immunosuppressive microenvironment that can impede the infiltration of immune cells, including CAR-T cells. The CXC motif chemokine receptor 5 (CXCR5) receptor, which guides cells to lymphoid tissues, might also assist CAR-T cells in navigating the complex stromal architecture of solid tumors, potentially leading to improved tumor invasion. This is why CAR-T cells co-expressing CXCR5 were superior to conventional CAR-T cells in eradicating ligand-positive tumor cells (10, 11). CXC-motif chemokine ligand 13 (CXCL13), the exclusive ligand for the CXCR5 receptor, is expressed in OS cell lines and is positively correlated with CD8^+^ T cell recruitment at tumor sites (12). However, reaching tumor sites is only part of the challenge; ensuring survival and long-term persistence at these locations is equally crucial for effective malignant cell eradication. Building on the previous success in utilizing interleukin-7 (IL-7) co-expression to enhance CAR-T cell therapy efficacy in a prostate cancer model (8), the present study presents a significant step forward. With our novel approach, we constructed a unique NKG2D-based CAR with the aim of augmenting CAR-T cell trafficking and persistence against OS. This construct co-expressed CXCR5 and IL-7 (C5/IL7) within the conventional NKG2D-CAR backbone, addressing both challenges, i.e., effective tumor penetration and sustained presence.

## 2 Materials and methods

### 2.1 Cell lines

HEK293T, U-2 OS, 143B, and Jurkat cell lines were used in this study. U-2 OS, 143B, and Mg63 are the three common osteosarcoma cell lines, they all express the ligand MICA/B of NKG2D, as well as the ligand CXCL13 of CXCR5, which are suitable for our study. All cells were obtained from the American Tissue Culture Collection (ATCC, USA) and were maintained in our laboratory. HEK293T and 143B cells were maintained in Dulbecco’s modified Eagle’s medium (DMEM; Gibco Laboratories, Grand Island, NY, USA), U-2 OS cells were maintained in McCoy’s 5A medium (Procell, Wuhan, China), and Jurkat cells were maintained in RPMI 1640 medium (Gibco Laboratories). The culture media were supplemented with 10% fetal bovine serum (FBS; ABW, Uruguay) and 1% penicillin-streptomycin, creating an optimal growth environment referred to as the complete medium. Additionally, 143B cells were genetically modified to express luciferase through firefly-luciferase lentiviral transduction. The passage number of all cell lines was within 15.

### 2.2 CAR construction and lentivirus production

The second-generation CAR vector utilizing the pLL3.7 plasmid was constructed as described previously (8, 9, 13). The plasmid contained an EF-1α promoter, the human CD8α signal peptide, the NKG2D extracellular domain, the CD8α hinge, and transmembrane domains, and the cytoplasmic domain of 4-1BB fused with the CD3ζ cytoplasmic tail. Additional modifications were introduced to construct novel C5/IL7-CAR plasmid expressing human CXCR5 (aa 1-372) and IL-7 (aa 1-177) with the N-terminal secretion signals P2A or T2A cleavage peptides. For lentivirus packaging, HEK293T cells were seeded and cultured for 24 hours, followed by transfection on day 1 with the lentiviral vector and two helper vectors (psPAX2, pMD2.G) using a polyethyleneimine (PEI) system (Polysciences, Warrington, PA, USA). After a 48-hour incubation period, the supernatant was collected, filtered (0.45µm), and concentrated via ultracentrifugation (27000g, 4°C, 2 hours). The supernatant was removed, and an additional culture medium was added to the concentrated virus mixture. Aliquots were preserved at -80°C.

### 2.3 T-cell isolation and CAR-T cell preparation

Peripheral blood mononuclear cells (PBMCs) were isolated from the blood of healthy donors (Shanghai Blood Center, China) by the Ficoll-Paque PLUS gradient centrifugation method (HyClone, Logan, UT, USA). Primary T cells were positively selected with CD4 and CD8 microbeads, stimulated with Enceed™ (Genscript, Nanjing, China) for 48 hours, and subsequently cultured in X-VIVO 15 mediums (Lonza) supplemented with 5% FBS, 1% penicillin/streptomycin, and 200IU/mL human recombinant interleukin-2 (hIL-2, Seaform Biotech., Beijing, China). To prepare CAR-T cells, 1×10^6^ T cells were transduced with the respective lentiviruses at a multiplicity of infection (MOI) 10. These transduced cells were then expanded for 2 weeks at a density of 1×10^6^/mL.

### 2.4 Flow cytometry analysis

Flow cytometry analysis was performed according to the experimental needs. Surface staining involved a 30-minute incubation of cells with FACS antibodies, whereas, for intracellular staining, CAR-T cells were fixed and permeabilized using the BD Cytofix/Cytoperm Kit (BD Biosciences, Bergen County, NJ, USA) before staining. The gating strategies were based on forward vs scatter characteristics, followed by single-cell gating of live cells. Antibodies were purchased from Biolegend, BD Biosciences, and eBiosciences™ USA, as detailed in **Supplementary Table S1**. The data were acquired using a BD LSRfortessa or CantoII flow cytometer, and the acquired samples were analyzed with FlowJo software (version 10.8.1).

### 2.5 RT-PCR

Total RNA was extracted from CAR-T cells or cancer cells and subjected to reverse transcription for cDNA synthesis as described previously.14 The specific primers used for IL-7, CXCL13 and β-actin were as follows: IL-7 (forward 5’- TACAGTACGCATGTGAAAGTCA-3’, reverse 5’-AGGAAACACAAGTCATTCAGTTTT-3’), CXCR5 (forward 5’-CCTTGCCTTGCCAGAGATTC-3’, reverse 5’-TGTAGAGCATGGGGTTGAGG-3’), CXCL13 (forward 5’-TGGGGCAAACTCAAGCTTCT-3’, reverse 5’-GTCTGGGGATCTTCGAATGCTA-3’), and β-actin (forward 5’- GTACGCCAACACAGTGCTG-3’, and reverse 5’-CGTCATACTCCTGCTTGCTG-3’). The RT-PCR reaction cycles comprised pre-denaturation at 95°C for 5 minutes, followed by 30 cycles of denaturation at 95°C for 30 seconds, annealing at 60°C for 30 seconds, and extension at 72°C for 30 seconds. The PCR product was loaded on 2% agarose gel, and electrophoresis was performed.

### 2.6 Transwell assay

CAR-T cells (3×10^5^) were seeded in the upper chamber of a 24-well transwell plate (3µm pore size, Corning), and the lower chamber was filled with DMEM (500µl) or tumor-cell supernatant. After a 4-hour incubation period, cells were collected from the lower chamber and quantified using a hemocytometer.

### 2.7 Cytotoxicity assay

3×104 luciferase-transduced target cells (U-2 OS, 143B, and Mg63) were seeded in a low-attachment 96-well plate. Effector cells, including Mock-T, CAR-T, and C5/IL7-CAR-T cells, were cultured at various effectors to target (E: T) ratios (1.25:1, 2.5:1, 5:1). After16-hours of coincubation, the cells were collected, and killing efficiency was quantified using a firefly Luciferase Reporter Gene Assay Kit (Beyotime Biotech, Shanghai, China).

### 2.8 Cell proliferation assay

A total of 1×10^6^ T cells from each group were labeled with carboxyfluorescein succinimidyl ester (CFSE; 65-0850-84, eBioscience™, Waltham, MA, USA) at a concentration of 10µM. The cells were incubated in the dark at 37°C for 15 minutes, after which the unbound CFSE was removed through FBS quenching by incubating the mixture at 4°C for 10 minutes. After washing, cells were resuspended, and baseline CFSE fluorescence was analyzed on day 0 using flow cytometry. Subsequently, the labeled cells were cultured for 5 days, and post-culture CFSE dilution was assessed via flow cytometry.

### 2.9 Enzyme-linked immunosorbent assay

In a controlled *in vitro* setting, CAR-T cells (1.5×10^5^) were cultured either alone or cocultured with 5×10^4^ tumor cells in a complete X-VIVO medium. After 24 hours, the cell-free supernatant was collected, and IL-7 protein secretion was quantified using an ELISA kit following the manufacturer’s instructions (1110702, Dakewe Biotech, Beijing, China). For *in vivo* assessment, blood samples were aseptically collected from mouse orbits into EDTA-coated EP tubes. After centrifugation at 3000rpm for 10 minutes, the supernatant was collected. Ferritin levels in the supernatant were assessed with a ferritin-ELISA kit according to the manufacturer’s instructions (U96-3528E, YoBiBiotech, Shanghai, China), and the measurements were performed using a microplate spectrophotometer (450nm).

### 2.10 *In vivo* study

All animal experiments were performed with the approval of the Animal Ethics Committee of East China Normal University. Eight-week-old female NOD/SCID/γ-chain-/- (NSG) mice were used to establish osteosarcoma xenografts. Mice were anesthesia using isoflurane at 0.41ml/min fresh gas flow (2% concentration). Initially, 2×10^6^ 143B luciferase-positive cancer cells were subcutaneously injected into the right flank of mice. After 2 weeks, when the tumor size reached 100-150mm3, the mice were randomly divided into 6 groups (n=6), and IVIS imaging was performed. Prior to each session, the mice were injected with D-luciferin (3mg/mouse) and subsequently sedated. The photons emitted from luciferase-expressing cells were quantified using Living Image software (version 4.4). The photon intensity is represented by red (high) to blue (low), providing insight into tumor progression. Furthermore, each group either received PBS, Mock-T (5×10^6^), CAR-T (5×10^6^), or 3 different dosages of C5/IL7-CAR-T (2×10^6^, 5×10^6^ and 1×10^7^) cells intravenously, followed by IVIS imaging every week. Tumor volume was regularly assessed using calipers with the formula V=1/2(length × width^2^). Peripheral blood was collected from mouse orbits on days 3, 12, and 23 after CAR-T cell therapy in EDTA tubes. Serum isolation was followed by cytokine (IL-8, IFN-γ) detection using a Cytometric Bead Array (CBA) kit (Dakewe Biotech., Beijing, China), and ferritin was detected through an ELISA kit (U96-3528E, YoBiBiotech, Shanghai, China). On day 27, the mice were sacrificed, and tumor and spleen tissues were collected for single-cell suspension preparation. Various markers were analyzed via flow cytometry, employing the antibodies specified in **Supplementary Table 1.**

### 2.11 Statistical analysis

GraphPad Prism (version 9.0) was used to generate the graphs and conduct the statistical analysis. The data are presented as the mean and standard deviation (± SD). The choice of statistical tests, including one-way ANOVA, two-way ANOVA, and two-tailed unpaired T-tests, was determined by the context of the analysis. Statistically significant differences are denoted as *P < 0.05, **P < 0.01, ***P < 0.001, ****P < 0.0001 and ns is non-significant.

## 3 Results

### 3.1 Construction and characterization of novel CAR

**Figure 1:**
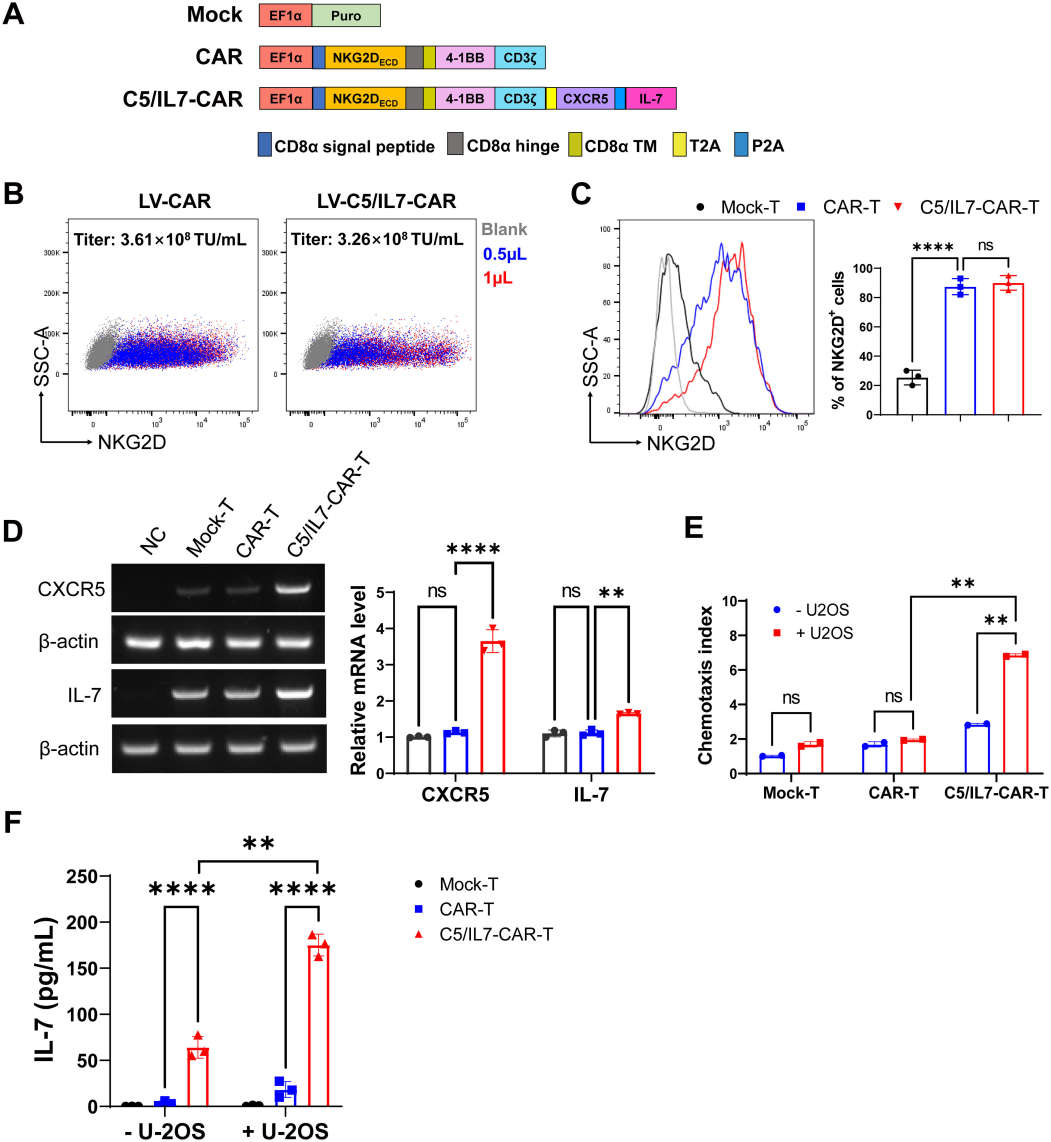
C5/IL7-CAR vector construction and validation. **(A)** Schematic representation of Mock, CAR, and C5/IL7-CAR constructs. **(B)** 2×10^5^ HEK293T cells were transduced with different volumes of CAR and C5/IL7-CAR lentiviruses separately. After 48 hours, NKG2D expressions were analyzed by flow cytometry. The percentages of positive cells indicated in the dot plot and the titer (TU/mL) were calculated with the formula: (2×10^5^×percentages of positive cells×1000)/Volume of lentiviruses. **(C)** 1×10^6^ primary T cells were transduced with Mock, CAR, and C5/IL7-CAR lentiviruses, MOI=10. After 48 hours, cells were analyzed by flow cytometry for NKG2D expression. The percentages of positive cells are indicated in the histogram (left panel) and bar chart (right panel). **(D)** Mock-T, CAR-T, and C5/IL7-CAR-T were collected 48 hours post-transduction and were subjected to RT-PCR for CXCR5 and IL-7 gene expression analysis. HEK293T cells were used as a negative control. β-actin was used as a reference housekeeping gene. **(E)** The Chemotaxis index of CAR-T cells was determined using a transwell assay. In each experimental group, 3×10^5^ cells were placed in the upper chamber of a 24-well transwell plate. After a 4-hour co-culture period, their migratory response towards the lower chamber containing either DMEM or supernatant from U-2 OS cells was evaluated. The number of migrated T cells was quantified using a hemocytometer. Data are presented based on three technical repeats with T cells cultured without cancer cell supernatant indicated in blue and those exposed to cancer cell supernatant in red. **(F)** Mock-T, CAR-T, and C5/IL7-CAR-T cohorts were cultured with or without U-2 OS cells for 24 hours, and the medium was collected and subjected to ELISA for IL-7 protein expression. Experiments were performed independently at least 3 times. For **(C)**, One-way ANOVA was used in Tukey’s multiple comparison test, and for **(D–F)**, Two-way ANOVA was used in Šídák’s multiple comparison test. Data represent Mean ± SD. **P < 0.01, ****P < 0.0001, ns, not significant.

The second-generation chimeric antigen receptor was used in this study as described in previously published data (8, 9, 13). Briefly, our CAR consisted of an NKG2D-based extracellular domain along with 4-1BB and CD3ζ intracellular domains. The interaction between the extracellular and intracellular domains was facilitated by the presence of the CD8α hinge and transmembrane domains. Additionally, a novel CAR was constructed by incorporating the coding sequences (CDSs) of the CXCR5 and IL-7 genes into the basic CAR framework; this construct was termed C5/IL7-CAR. A schematic representation is shown in **Figure 1A**. The puromycin lentiviral vector was used as a control and was termed Mock.

HEK293T cells were co-transfected with either CAR or C5IL7-CAR in the presence of three plasmid packaging systems, as described previously (14, 15). The lentivirus was collected 48 and 72 h post-transfection, and titer was calculated. The lentivirus for both CAR-plasmids gave a high infection rate in HEK293T cells, giving excellent titer (**Figure 1B**). Activated T cells were transduced with Mock, CAR, or C5/IL7-CAR lentivirus to generate Mock-T, CAR-T, or C5/IL7-CAR-T cells, respectively. Subsequently, cells were stained with an anti-human NKG2D antibody 48 hours post-transduction. Since NKG2D is part of the CAR design, its expression directly reflects the incorporation of the CAR into the T cell population. Monitoring NKG2D expression provides insight into the extent to which engineered cells express the intended chimeric receptor. Both CAR T cells displayed notable transduction efficiency compared to that of Mock-T (**Figure 1C**).

To validate the efficiency of our novel C5/IL7-CAR construct, the expression levels of CXCR5 and IL-7 were determined in successive experiments. The RT-PCR data indicated that C5/IL7-CAR-T cells demonstrated higher gene expression for CXCR5 and IL-7 genes as compared with the wild-type CAR-T cells (**Figure 1D**). The insertion of CXCR5 and IL-7 into CAR plasmid was further confirmed by transwell assay and ELISA, respectively. The increase in the chemotaxis index in C5/IL7-CAR-T cells observed by the transwell assay confirmed the successful insertion of the CXCR5 CDS (**Figure 1E**). Our ELISA data indicated that IL-7 expression was elevated significantly in C5/IL7-CAR-T cells in the absence of target cells. In the presence of U-2 OS cells, IL-7 expression was further amplified (**Figure 1F**). These data confirmed the successful generation and functioning of the novel C5/IL7-CAR vector.

### 3.2 C5/IL7 amplified CAR-T cell cytotoxicity *in vitro*

**Figure 2:**
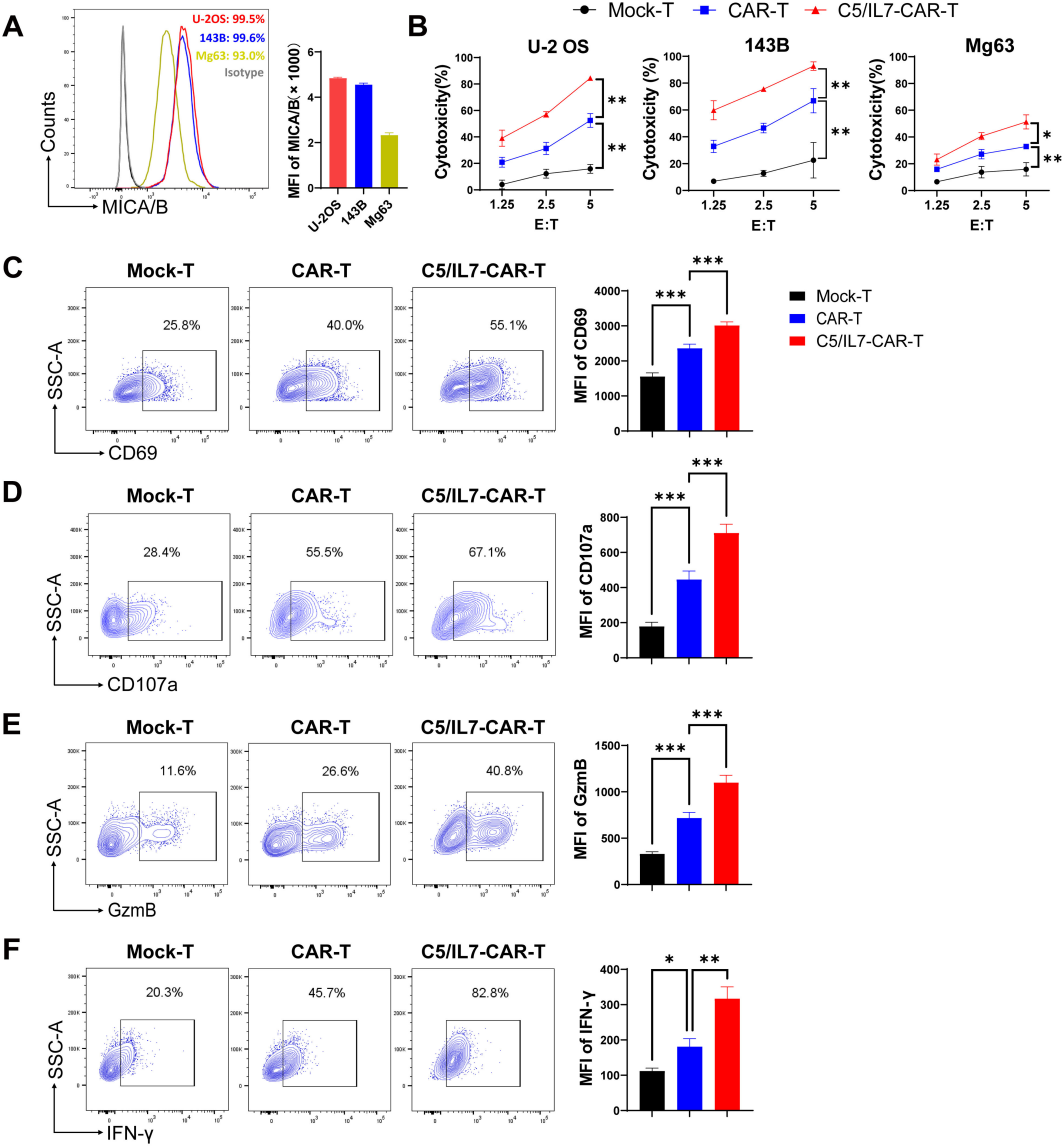
*In vitro* effector function of C5/IL7-CAR-T cells. **(A)** U-2 OS, 143B, and Mg63 cells were analyzed by flow cytometry for NKG2D Ligands (MICA/B) expression. U-2 OS is shown in red, 143B in blue and Mg63 in the yellow color histogram. Data are presented as Mean Fluorescence Intensity (MFI) normalized to unstained cells. **(B)** Cytotoxic analysis against U-2 OS, 143B, and Mg63 target cells. Experimental cohorts were cocultured with luciferase-transduced U-2 OS, 143B, and Mg63 target cells at varying effector-to-target (E: T) ratios for 16 hours. Percentage lysis was quantified using a luciferase-based cytotoxicity assay. **(C–F)** Flow cytometry analysis of CD69, CD107a, GzmB, and IFN-γ expression in Mock-T, CAR-T, and C5/IL7-CAR-T cohorts following a 16-hour co-culture with target cells at an E: T ratio of 3:1. Data is presented in a contour plot (left panel), and MFI is indicated by bar graphs (right panel). Experiments were performed independently at least 3 times. One-way ANOVA was used in Tukey’s multiple comparison test, and Data represent Mean ± SD. *P < 0.05, **P < 0.01, ***P < 0.001.

To determine the suitability of osteosarcoma as a potential target for NKG2D-based CAR-T cell therapy, MICA/B expression in human OS cell lines was determined by flow cytometry. We found that OS cell lines U-2 OS, 143B, and Mg63 had increased expression of NKG2D ligands on their surface, suggesting that these cells could be suitable targets for our novel NKG2D-based CAR construct (**Figure 2A**). Previous data suggest increased expression of CXCL13, the ligand for CXCR5, in OS (16, 17), which was further confirmed in the present study (**Supplementary Figure 1**). Given the high expression of MICA/B and CXCL13, human osteosarcoma cell lines constitute the perfect avenue for studying our CAR system expressing NKG2D, CXCR5, and IL-7. To evaluate the cytotoxicity of experimental groups, we performed cytotoxicity assays targeting U-2 OS, 143B, and Mg63 transgenic cell lines as targets (**Figure 2B**). C5/IL7-CAR-T cells exhibited enhanced killing capacity against OS cell lines compared to that of wild-type CAR-T cells. We conducted a series of flow cytometry experiments to further investigate the effects of C5/IL7 co-expression on CAR-T cell activation, degranulation, and cytokine release. Mock-T, CAR-T, and C5/IL7-CAR-T cells were cocultured with U-2 OS for 16 hours. Subsequently, the cells were stained with C-type lectin domain (CD69) and lysosomal-associated membrane protein 1 (CD107a) flow cytometry antibodies to assess T cell activation and degranulation, respectively. C5/IL7 incorporation improved CAR-T cells’ activation and degranulation capacity (**Figures 2C, D**). Increased CD69 expression suggested that transduced T cells are more responsive to the presence of cancer cells. Improved degranulation and activation advocate that CAR-T cells are more effective in enhancing the antitumor response (18). Furthermore, cells that exhibit increased CD107a expression are more effective at releasing cytotoxic granular contents (9). Therefore, we further analyzed the levels of intracellular granzyme B (GzmB) and interferon-gamma (IFN-γ) in the experimental cohorts by flow cytometry. As expected, C5/IL7-CAR-T cells expressed more GzmB and IFN-γ than did the wild-type CAR-T cells (**Figures 2E, F**). The data above was further confirmed in the presence of 143B target cells (**Supplementary Figure 2**). The results signify that incorporating C5/IL7 enhances the cytotoxic potential of CAR-T cells against osteosarcoma cell lines. This augmentation is likely achieved by increased activation, degranulation, and cytotoxic molecules and cytokines release.

### 3.3 C5/IL7 enhanced CAR-T cell survival

**Figure 3:**
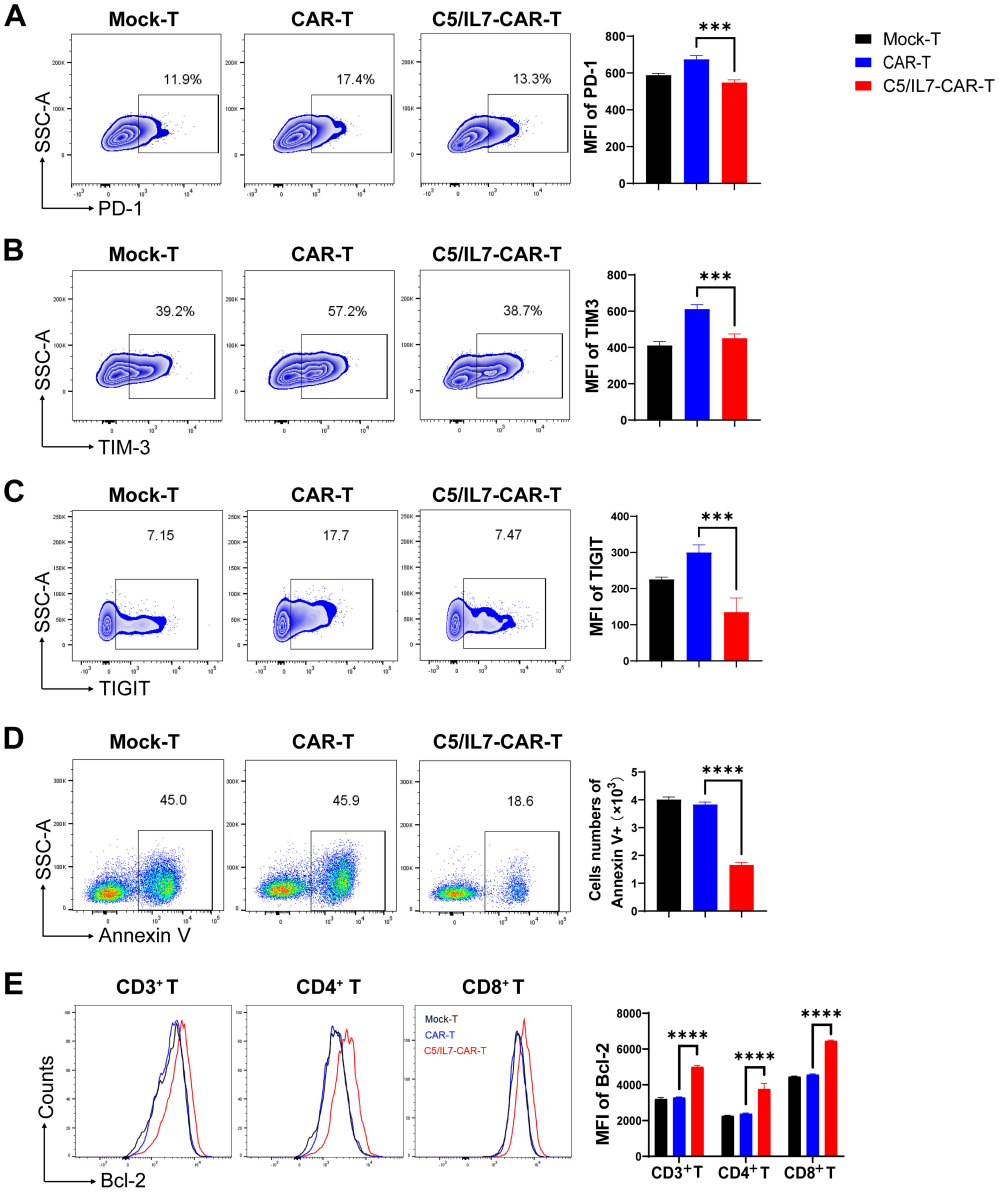
C5/IL7-CAR-T cells displayed reduced exhaustion and apoptosis *in vitro*. **(A–C)** Flow cytometry analysis of exhaustion markers PD-1, TIM-3, and TIGIT was performed after co-culturing Mock-T, CAR-T, and C5/IL7-CAR-T cells with U-2 OS for 3 days at an E: T ratio of 3:1. Representative FACS files are shown on the left, and MFI bar graph is presented on the right side. **(D)** Flow cytometry-based quantification of apoptotic cells in Mock-T, CAR-T, and C5/IL7-CAR-T cohorts. The cells were cultured for 5 days, harvested, and subsequently stained with Annexin V for 15 minutes in the dark at room temperature (RT). Flow cytometry analysis was conducted. The left and right panels displayed representative FACS files and absolute Annexin V^+^ cell counts, respectively. **(E)** Following a 5-day culture of Mock-T, CAR-T, and C5/IL7-CAR-T cells were collected, and Bcl-2 expression within CD3^+^, CD4^+^, and CD8^+^ T-cell populations was evaluated using flow cytometry. Representative histogram (left panel) and MFI (right panel) are shown. Experiments were performed independently at least 3 times. For **(A–D)**, One-way ANOVA was used in Tukey’s multiple comparison test; for **(E)**, Two-way ANOVA was used in Tukey’s multiple comparison test. Data represent Mean ± SD. ***P < 0.001, ****P < 0.0001.

To explore the potential of co-expressing CXCR5 and IL-7 to reduce the exhaustion and apoptosis of CAR-T cells, we performed flow cytometry-based assays by staining cocultured cells with antibodies related to the respective phenomenon. The experimental groups were cocultured with target cells for 3 days. Subsequently, the cells were washed with PBS to remove traces of culture medium and stained with anti-human programmed cell death protein 1 (PD-1) and anti-human T cell immunoglobulin and mucin domain 3 (TIM-3) flow cytometry antibodies. FACS analysis revealed that C5/IL7-CAR-T cells had significantly lower levels of PD-1 and TIM-3 compared to the CAR-T group (**Figures 3A, B**). Moreover, the percentage of PD-1/TIM-3 double-positive cells was reduced in the novel-CAR transduced T cells (**Supplementary Figure 3A**). Similarly, T cell immunoglobulin and ITIM domain (TIGIT) expression was also decreased in CXCR5 and IL-7 expressing CAR-T experimental group (**Figure 3C**). In the absence of target cells, CXCR5 and IL-7 co-expression prevented CAR-T cells from undergoing IL-2-mediated exhaustion in long-term culture conditions (**Supplementary Figures 3B-E**). Decreased expression of exhaustion markers in CAR-T cells is beneficial because exhaustion can reduce immune responses against target cells (19). Although exhaustion and apoptosis are distinct processes, they can also be interrelated. Continued exhaustion of T cells may ultimately lead to their apoptosis as they become functionally impaired and unskilled to elicit effective immune responses. The expression of inhibitory receptors can also modulate apoptotic signaling pathways, possibly enhancing the susceptibility of exhausted T cells to cell death (20, 21). In the present study, reduced exhaustion of C5/IL7-CAR-T cells may have contributed to decreased apoptosis (**Figure 3D**). Flow cytometry data indicated that C5/IL7-CAR-T cells exhibited decreased Annexin V and increased B-cell lymphoma 2 (Bcl-2) expression. Bcl-2 was significantly elevated in CD4^+^ T cells and CD8^+^ T cells within the CD3 gate in the C5/IL7-CAR-T cohort (**Figures 3D, E**). These results collectively demonstrated that novel CAR-T cells are more resistant to exhaustion and apoptosis than their wild-type counterparts are.

### 3.4 C5/IL7 augments CAR-T proliferation and persistence

**Figure 4:**
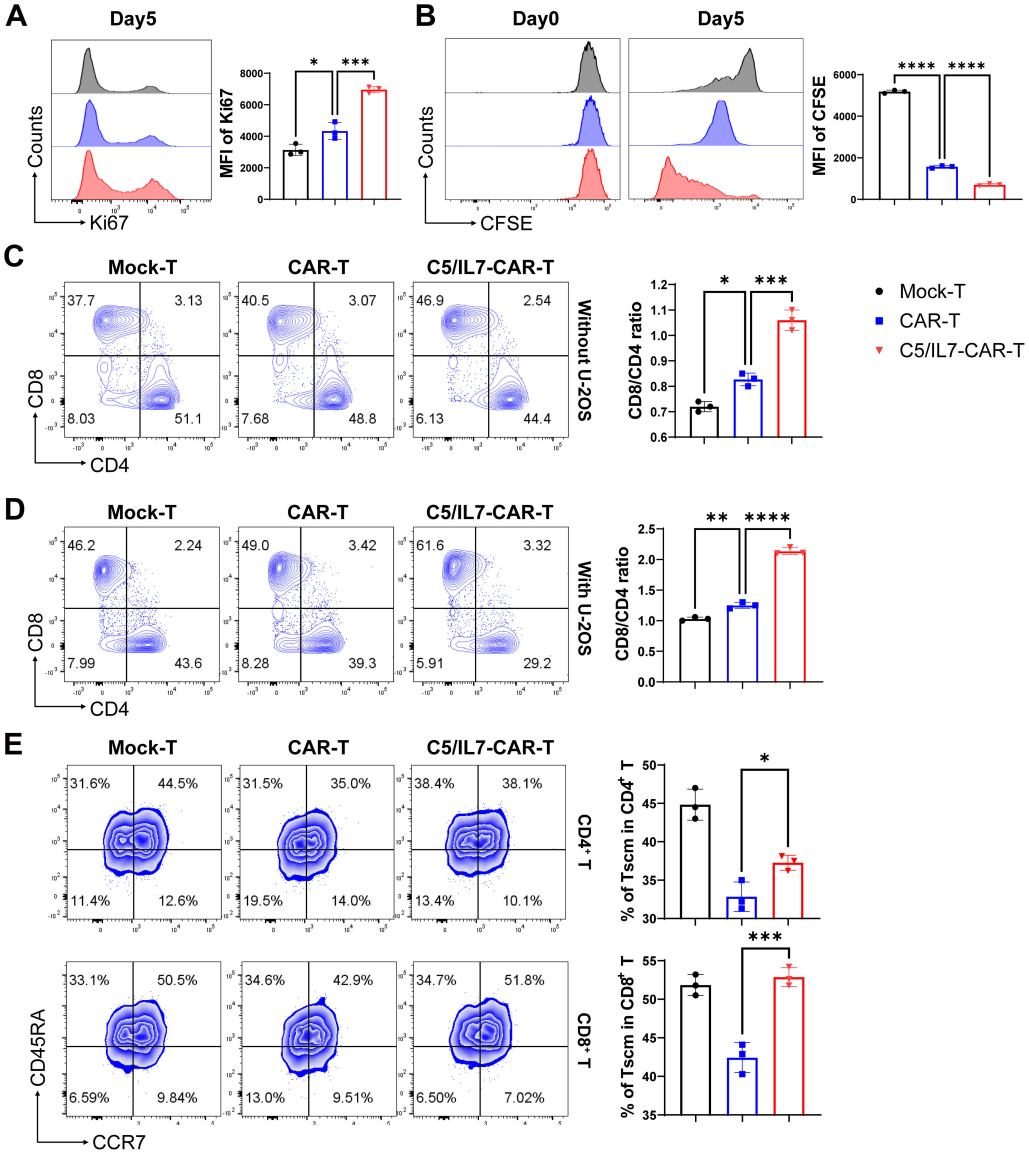
C5/IL7-CAR-T cells exhibit enhanced proliferation and differentiation. **(A)** To assess proliferation in the absence of target cells, Mock-T, CAR-T, and C5/IL7-CAR-T cells were cultured for 5 days, and analysis of Ki67 expression was detected by flow cytometry. A representative histogram is shown on the left, and MFI is presented on the right side. **(B)** To further assess proliferation in the presence of target cells, 1×10^6^ cells from Mock-T, CAR-T, and C5/IL7-CAR-T groups were initially labeled with 1µl of CFSE, and 2×10^5^ cells underwent flow cytometry analysis for CFSE dilution designated as day 0 in FITC channel. The remaining cells were co-cultured with U-2 OS for 5 days. Subsequently, on the 5th day, the CFSE dilution rate was once again measured using the same template in flow cytometry. **(C, D)** Mock-T, CAR-T, and C5/IL7-CAR-T cells were cultured with or without U-2 OS for 5 days. Flow cytometry analysis determined T cell subsets, presented in the left panel, while the calculated CD8/CD4 ratio is shown in the right panel. **(E)** For T cell differentiation, cells from each experimental group, after 5 days of co-cultured with U-2OS, were analyzed by flow cytometry. Tscm: Stem cell memory T cell (CCR7^+^CD45RA^+^), Tcm: Central memory T cell (CCR7^+^CD45RA^-^), Tem: Effector memory T cell (CCR7^-^CD45RA^-^), Teff: Effector T cell (CCR7^-^CD45RA^+^). Experiments were performed independently at least 3 times. One-way ANOVA was used in Tukey’s multiple comparison test, and data represent Mean ± SD. *P < 0.05, **P < 0.01, ***P < 0.001, ****P < 0.0001.

Improved CAR-T cell proliferation can lead to a larger population of active cytotoxic T cells, resulting in a more effective response against cancer cells (22). Flow cytometry assays were conducted to evaluate the impact of C5/IL7 on CAR-T cell proliferation. Novel CAR-T cells demonstrated enhanced Ki67 expression than did the wild type CAR-T cells in the absence of target cells (**Figure 4A**). The proliferation phenomenon was further analyzed in the presence of target cells. The cells in the experimental cohorts were stained with CFSE and cultured with U-2 OS for 5 days. Flow cytometry analysis of CFSE dilution was conducted on days 0 and 5. Notably, compared with Mock-T and CAR-T cells, C5/IL7-CAR-T cells exhibited significantly greater proliferation rates (**Figure 4B**). Next, we investigated the differentiation of C5/IL7-CAR-T cells based on CD8 and CD4 expression. Mock-T, CAR-T, and C5/IL7-CAR-T cells were cultured in the presence or absence of target cells for 5 days and stained with CD8 and CD4 FACS antibodies. The CD8^+^/CD4^+^ T cell ratio is traditionally used to measure immune cell activity, and CD8+ T cells represent the mainstay of CAR-T cell therapy (23). CAR-T cells co-expressing C5/IL7 increased the CD8/CD4 ratio, as demonstrated by flow cytometry (**Figures 4C, D**). An increased CD8/CD4 ratio indicated that the percentage of cytotoxic T lymphocytes was improved compared to that of helper T lymphocytes. To determine whether C5/IL7 influences the maintenance of stemness and memory properties, Mock-T, CAR-T, and C5/IL7-CAR-T cells were analyzed on the basis of CD45RA and CCR7 expression within CD4^+^ and CD8^+^ T cell gates in the presence of U-2 OS target cells. Remarkably, compared with wild-type CAR-T cells, C5/IL7-CAR-T cells preserved a greater percentage of Stem cell memory T cells (Tscm) (**Figure 4E**). The results were confirmed in the 143B OS cell line (**Supplementary Figure 4**). We also analyzed on the basis of CD45RO and CD62L expression within CD4^+^ and CD8^+^ T cell gates in the absence of target cells (**Supplementary Figure 5**). The increased proportions of Tscm cells in the C5/IL7-CAR-T cell cohort suggest that these cells may have a longer lifespan and will remain active over an extended period of time. This potential longevity could provide sustained tumor control and reduce the risk of relapse. Overall, the results demonstrated that incorporating C5/IL7 into CAR can enhance transduced T cells’ proliferative potential and lead to the development of phenotypes well-suited for successful cancer immunotherapy.

### 3.5 C5/IL7 improves CAR-T therapy efficacy *in vivo*

**Figure 5:**
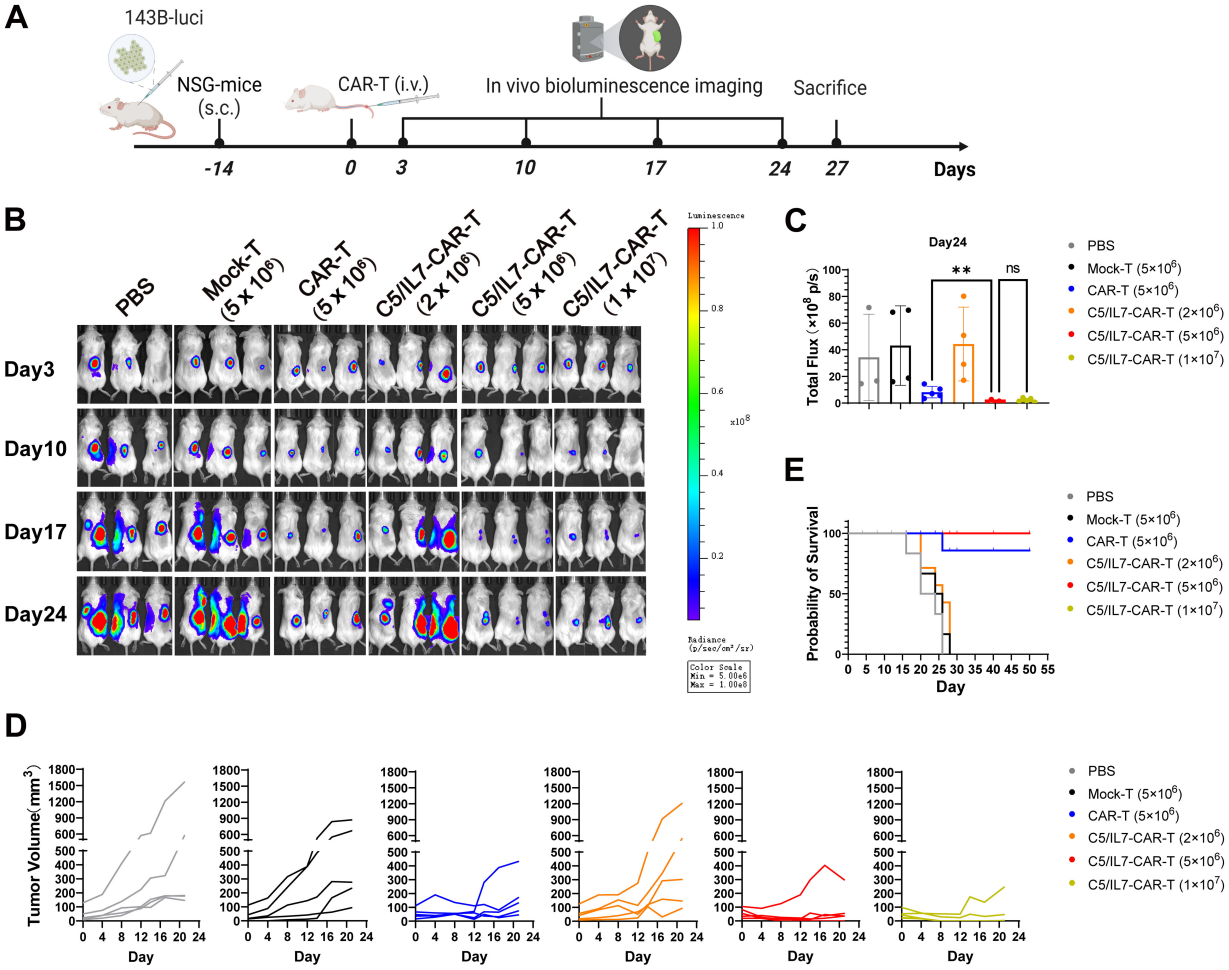
C5/IL7-CAR-T cells express advanced and enhanced antitumor activity *in vivo*. **(A)** Schematic representation of *in vivo* experimental design. NSG mice were subcutaneously (s.c.) inoculated with 2×10^6^ 143B-luciferase cells/mouse; Mice were randomly divided into 6 experimental groups (n=6), receiving treatments of PBS, Mock-T (5×10^6^), CAR-T (5×10^6^), and C5/IL7-CAR-T with varying cell numbers (2×10^6^, 5×10^6^, 1×10^7^) cells/mice. **(B)** *In vivo*, bioluminescence imaging was employed to assess the progression of 143B-Luciferase+ tumors in each experimental group on days 3, 10, 17, and 24 (n=5). **(C)** On day 24, the total bioluminescence flux (photons per second) emitted by tumors was quantified through bioluminescence imaging. **(D)** Tumor volume was assessed at 3-day intervals using calipers. Each line on the graph represents the dynamic representation of tumor volume for an individual mouse (n=5). **(E)** Kaplan-Meier survival curve visually depicted the overall survival data for mice in each experimental group (n=6). Statistical significance was assessed using one-way ANOVA Tukey’s test. **p < 0.01 and ns is non-significant.

The outcomes of our *in vitro* experiments clearly demonstrated the superior performance of the C5/IL7-CAR-T cells compared to conventional CAR-T cells. CAR-T cells that co-expressed C5/IL7 exhibited heightened activation, degranulation, cytokine release, and, ultimately, enhanced cytotoxicity against OS cell lines. Next, to determine the ability of the experimental cohorts to eradicate tumors *in vivo*, we inoculated 143B cells into NSG mice. After 14 days, the mice were randomly divided into 6 groups. The PBS, Mock, or CAR transduced T cells were injected intravenously (i.v.) through a tail vein into the respective groups of mice. The imaging of the implanted tumors was carried out with an IVIS every few days (**Figure 5A**). The CAR-T cell-treated mice receiving five million cells/mouse were able to control tumor growth as compared to the Mock-T cell and PBS. The C5/IL7-CAR-T did not provide significant tumor control at a dose of 2 million cells per mouse. C5/IL7-CAR-T cells had better antitumor efficacy than conventional CAR-T cells at the same dose. Furthermore, doubling this dose did not improve tumor control (**Figures 5B-D**; **Supplementary Figures 6**, **7**), yet the mice survived until the last observation day (Day 50) (**Figure 5E**). Previously, several safety concerns were raised in NKG2D-based CAR-T cell systems in OS xenograft models (7, 24). The results of ferritin, IFN-γ, and IL-8 analysis in the blood of experimental mice further support the safety of NKG2D-based CAR-T cell therapy in the OS model (**Supplementary Figure 8**). The results here demonstrated that in human OS mouse xenografts, mice receiving novel CAR-T cells outclassed wild-type CAR-T cells. The enhanced killing capacity of novel CAR-construct *in vivo* might correspond to the enhanced migration/tumor infiltration as well as survival ability of transduced T cells.

### 3.6 C5/IL7 improves CAR-T cell numbers and activities *in vivo*

**Figure 6:**
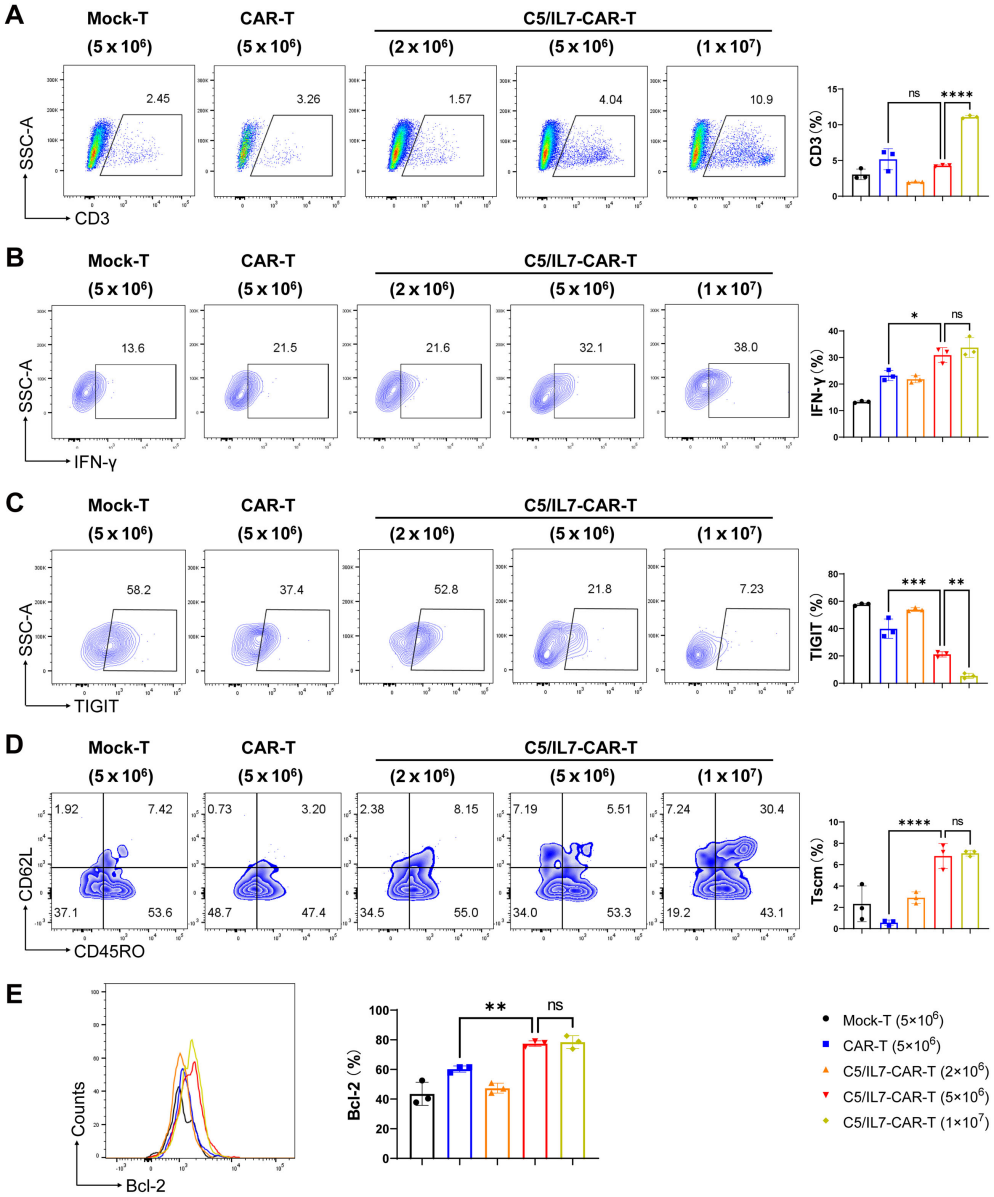
C5/IL7-CAR-T cells displayed better migration and survival *in vivo*. Mice were sacrificed on day 27 post-treatment; tumors were extracted, and single-cell suspensions were prepared for detailed immuno-profiling using flow cytometry analysis (n=3). The assessment encompassing: **(A)** CD3^+^ T cells were quantified through staining with BV421 anti-human CD3 antibody, **(B)** IFN-γ levels were assessed by staining with APC anti-human IFN-γ antibody, **(C)** TIGIT expression was examined by BV421 anti-human TIGIT antibody, **(D)** T cell differentiation status was characterized through staining with PE anti-human CD45RO, AF700 anti-human CD62L and BV421 anti-human CD3 antibodies. **(E)** Bcl-2 expression was analyzed after intracellular staining with PE anti-human Bcl-2 antibody. Each experiment was conducted independently. FACS analysis files are presented in the left panel, while the corresponding column chats on the right. Statistical significance was measured using way one-way ANOVA. *p < 0.05, **p < 0.01, ***p < 0.001, ****p < 0.0001 and ns is non-significant.

Next, we wanted to determine the quantity and quality of the T lymphocytes (TILs) in experimental groups. We sought to assess the impact of co-expressing C5/IL7 in CAR-T cells. After staining with anti-human CD3 FACS antibodies and subsequent flow cytometry analysis, we observed a substantial increase in CD3+ cells within the tumor of the C5/IL7-CAR-T receiving mice group, as shown in **Figure 6A**. Similar results were obtained for splenic CD3+ T cells (**Supplementary Figure 9**). The mechanism underlying this effect might be rooted in the homing properties of CXCR5, which facilitate the directed migration of T cells (11, 25, 26). Additionally, functional analysis revealed that the C5/IL7-CAR-T cells exhibited superior cytokine production, particularly IFN-γ release, compared to that of wild-type CAR-T cells (**Figure 6B**). Moreover, FACS analysis revealed a notable decrease in exhaustion (**Figure 6C**) and an increase in the proportion of Tscm populations within the CD3 gate (**Figure 6D**; **Supplementary Figure 10**). Our novel CAR construct improved the survival of transduced T cells inside the tumor microenvironment (**Figure 6E**). The data in this figure explicitly demonstrate the superior performance of C5/IL7-CAR-T cells in terms of numbers at the tumor site, cytokine release, and survival in a murine model. The combination of CXCR5 and IL-7 co-expression in CAR-T cells not only augments quantity but also enhances the quality of T cells, thereby contributing to an enhanced antitumor immune response in the OS microenvironment (27).

### 3.7 C5/IL7 regulated CAR-T cell function via pSTAT5 signaling

**Figure 7:**
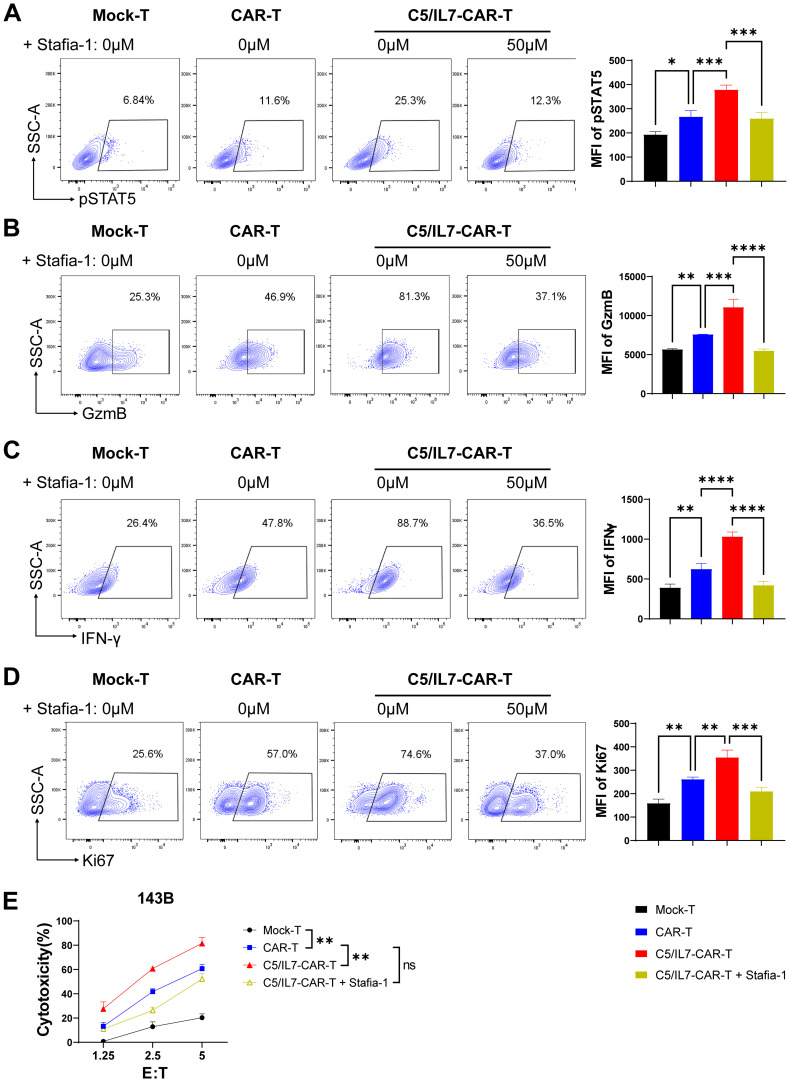
C5/IL7-CAR-T cells exhibit antitumor activity via pSTAT5 signaling. **(A–D)**, Mock-T, CAR-T, and C5/IL7-CAR-T cells, treated with either DMSO or Stafia-1 (50µM), were co-incubated with 143B cells for 24 hours at an E: T ratio of 3:1. Cells were then stained with FACS antibodies to detect pSTAT5 **(A)**, GzmB **(B)**, IFN-γ **(C)** and Ki67 **(D)** expression using flow cytometry. Representative FACS files are indicated on the left, and MFI bar graphs are shown on the right. **(E)**, Mock-T, CAR-T, C5/IL7-CAR-T and Stafia-1 treated C5/IL7-CAR-T cells were co-cultured with 143B-luciferase cells at various E: T ratios (1.25:1, 2.5:1 and 5:1) for 16-hours. Cytotoxicity was quantified using a luciferase assay. Experiments were performed independently at least 3 times. One-way ANOVA was used in Tukey’s multiple comparison test, and data represent Mean ± SD. *P < 0.05, **P < 0.01, ***P < 0.001, ****P < 0.0001, ns, ns, not significant.

To better understand the underlying molecular mechanisms, we focused on the STAT5 signaling pathway, which is known for its role in T cell functions. Flow cytometry data revealed that, compared with wild-type CAR transduced cells, C5/IL7-CAR transduced cells exhibited increased expression of phosphorylated STAT5 both in primary T cells and Jurkat cells. (**Figure 7A**; **Supplementary Figure 11**). Inhibition of STAT5 signaling in the C5/IL7-CAR cohort by Stafia-1 reversed the observed increase in STAT5 expression to a level equivalent to that of CAR-T cells (**Figure 7A**). To determine the role of STAT5 signaling in enhancing the functionality of C5/IL7-CAR-T cells, we employed a STAT5 inhibitor and detected different functional markers in the experimental cohorts via flow cytometry. Stafia-1 treatment decreased the levels of GzmB, INF-γ, and Ki67 markers in C5/IL7-CAR-T cells (**Figures 7B-D**). Moreover, the enhanced cytotoxic ability of C5/IL7-CAR-T cells was also diminished in the presence of the Stafia-1, as determined by cytotoxicity analysis (**Figure 7E**). These results underscore the critical role of C5/IL7 incorporation in CAR-T cell functionality.

## 4 Discussion

CAR-T cell therapy has achieved significant success in the treatment of blood cancers, yet its effectiveness in solid tumors has been constrained. While several factors contribute to this issue, the limited infiltration and survival of both CAR-T cells and native immune cells within the tumor microenvironment play fundamental roles in suboptimal antitumor immunity (28). In the current study, we utilized pre-synthesized CD3ζ and 4-1BB-based second-generation NKG2D-CAR systems. Additionally, we constructed a C5/IL7-CAR and compared its immunotherapeutic potential with that of conventional CAR. It was reported in some studies (although negated by others) that the second-generation CAR system outperformed the third-generation CAR system by providing a more potent antitumor response under certain circumstances (29, 30). The fact that CXCR5 and IL-7 were supposed to improve CAR-T cell numbers and activities, respectively (8, 11, 31, 32), a novel CAR co-expressing C5/IL7 was constructed. The C5/IL7-CAR transduced T cells demonstrated heightened expression of both CXCR5 and IL-7.

Osteosarcoma is the most common cancer in children and teenagers. The pursuit of novel and effective targets for CAR-T therapy in OS remains ongoing. For example, during this exploration, HER2-directed CAR-T cells achieved excellent results in preclinical studies while others are undergoing clinical trials (10). Here, we report a high expression of MICA/B in human OS cell lines U-2 OS and 143B cells, which is consistent with previously reported data (33). That is why NKG2D-based CAR-T cells demonstrated enhanced killing of OS *in vitro* compared to Mock-transduced T cells. Interestingly, C5/IL7-CAR-T cells not only displayed superior migration but also outperformed previous approaches involving CXCR5 and EGFR-based CAR-T cells, where no improvement in *in vitro* cytotoxicity was observed (11). The increased cytotoxic potential of NKG2D-based CAR-T cells *in vitro* in the current study, together with enhanced activation (CD69), degranulation (CD107a and GzmB), and cytokine release (IFN-γ) induced by C5/IL7 co-expression might owe to IL-7 signaling (34). Importantly, our *in vivo* observations mirrored the *in vitro* findings, with consistent patterns of tumor control. The tumor volume in mice receiving C5/IL7-CAR-T cells was significantly smaller than that in mice treated with wild-type CAR-T cells, together with an increased number of CAR-T cells at the tumor sites.

The microenvironment of solid tumors is extremely hypoxic due to inadequate blood supply and overwhelmed oxygen demand (35). Additionally, nutrient deprivation and upregulation of coinhibitory ligands further complicate the picture. As a result, T cells in the tumor microenvironment become exhausted and are prone to apoptosis (3). The exhaustion phenomenon is characterized by the upregulation of checkpoint molecules, loss of ability to secrete effector cytokines, and decreased proliferative ability (36). Our data demonstrated that CAR-T cells coupled with CXCR5 and IL-7 reduced the expression of checkpoint molecules *in vitro*. Concomitantly, C5/IL7-CAR-T cells in the osteosarcoma microenvironment displayed low TIGIT expression and were able to express more IFN-γ in the NSG mouse model. Furthermore, increased expression of Bcl-2 in C5/IL7-CAR-T cells further suggested that these cells are more resilient and capable of sustaining their antitumor function than their wild-type counterparts are. C5/IL7-CAR-T cells exhibited significantly greater proliferation rates than did the other CAR-T cells, suggesting that C5/IL7-CAR-T cells more rapidly expanded in response to tumor cells. Moreover, these cells displayed an increased CD8/CD4 ratio and Tscm phenotype, indicating a more favorable immune cell composition for CAR-T cell therapy.

STAT5 signaling is important for the survival of T lymphocytes at tumor sites. Previous studies have demonstrated that an increased STAT5 signaling can improve immunotherapeutic outcomes and hence provide better antitumor control (13, 37). In the current study, C5/IL7-CAR-T cells exhibited heightened STAT5 expression than did conventional CAR-T cells. The beneficial effects of C5/IL7 incorporation into CAR-T cells regarding proliferation, cytokine release, and cytotoxicity were diminished in the presence of Stafia-1. This fully demonstrates the importance of the STAT5 signaling pathway for CAR-T function. In the tumor microenvironment, effector T cells are typically in a state of high exhaustion, and rapid proliferation and viability maintenance of CAR-T cells can compensate for the over-exhaustion and depletion of effector T cells, resulting in better tumor clearance (38). IL-7 is a key factor in T function, and when IL-7 binds to its receptor, it can activate the JAK1 and JAK3 proteins in the cell, thereby phosphorylating the STAT3 and STAT5 proteins, forming heterodimers to enter the nucleus, activate the expression of downstream genes, and enhance the function of T cells. We also tested the STAT3 signaling pathway, which was expressed at very low levels, demonstrating that IL-7 is a STAT5 signaling pathway that promotes CAR-T cell proliferation, differentiation, and anti-apoptosis (39).

Overall, the current study suggests that C5/IL7 co-expression improves the efficacy of CAR-T cell therapy for OS. This new strategy provides a dual benefit by increasing the number and viability of transduced T cells. However, some potential limitations of the basic research include tumor immune evasion, as well as tumor heterogeneity and spread. Further improvements are necessary for the full implementation of this innovative CAR-T cell therapy in clinical translations. We will further use the osteosarcoma orthotopic model and PDX model, in combination with immune checkpoint blockade therapy, for further preclinical validation. In addition, our laboratory has also made some progress in overcoming tumor microenvironment-mediated immunosuppression for CAR-T cell therapy (13, 14). Only by combining these we can achieve the clinical translation.

## 5 Conclusions

In conclusion, the co-expression of CXCR5 and IL-7 in NKG2D-CAR-T cells holds promise for optimizing CAR-T cell therapy for osteosarcoma. The combined evidence from our *in vitro* and *in vivo* experiments demonstrated the multifaceted benefits of this approach, including improved cytotoxicity, resilience, and long-term persistence, which are critical for the success of immunotherapy against solid tumors. Further research and clinical investigations are warranted to fully grasp this innovative CAR-T cell therapy’s potential.

## Data Availability

The original contributions presented in the study are included in the article/**Supplementary Material**. Further inquiries can be directed to the corresponding author.
