# Supplementary material for: A novel strategy of co-expressing CXCR5 and IL-7 enhances CAR-T cell effectiveness in osteosarcoma
Source: Front Immunol. 2024 Oct 10;15:1462076. doi: 10.3389/fimmu.2024.1462076 (PMC11499113; doi:10.3389/fimmu.2024.1462076)
Supplement: Supplementary file 1 [file DataSheet1.docx]

Supplementary Material

**Supplementary Figures**

**
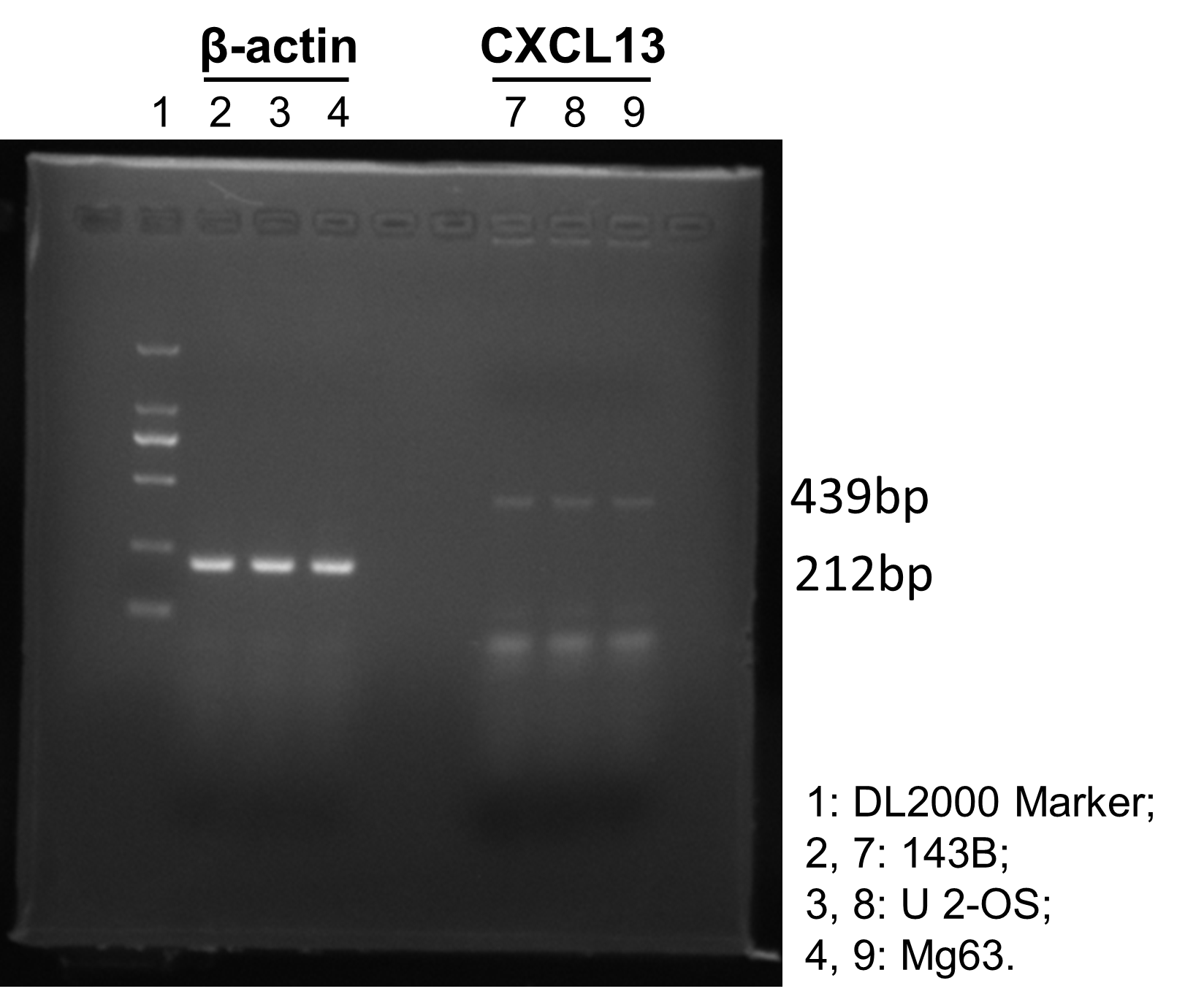
**

**Supplementary Figure 1.** The gene expression analysis of CXCL13 in 143B, U-2 OS, and Mg63 osteosarcoma cell lines by RT-PCR. Lane 1 represents the DNA marker of 2000bp, and β-actin was used as a reference gene.


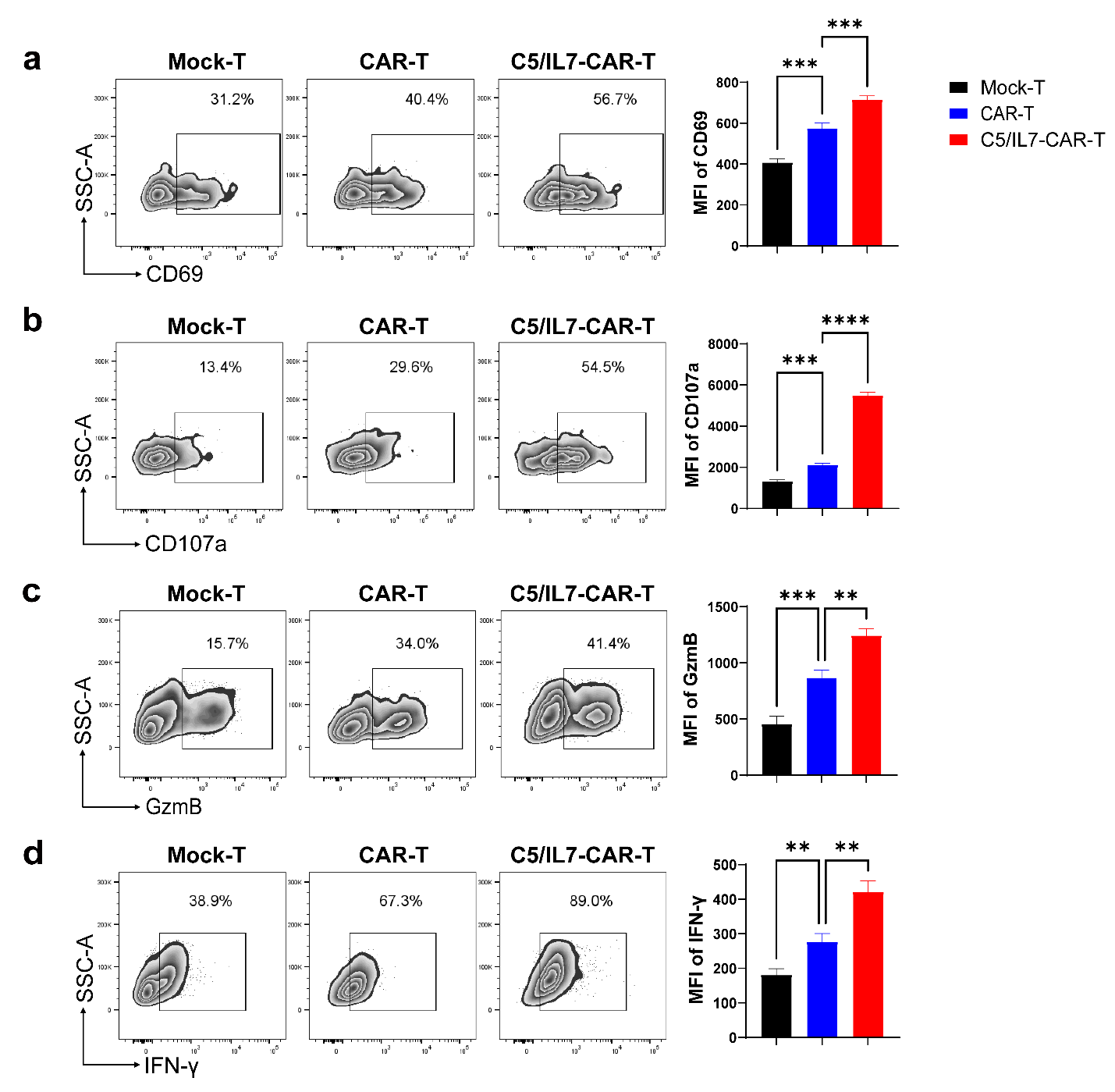


**Supplementary Figure 2.** C5/IL7 increases activation, degranulation, and cytokine release against the 143B OS cell line. Flow cytometry analysis of (a) CD69, (b) CD107a, (c) GzmB, and (d) IFN-γ. Experiments were performed independently at least 3 times. One-way ANOVA was used in Tukey’s multiple comparison test, and data represent Mean ± SD. **P < 0.01, ***P < 0.001, ****P < 0.0001.


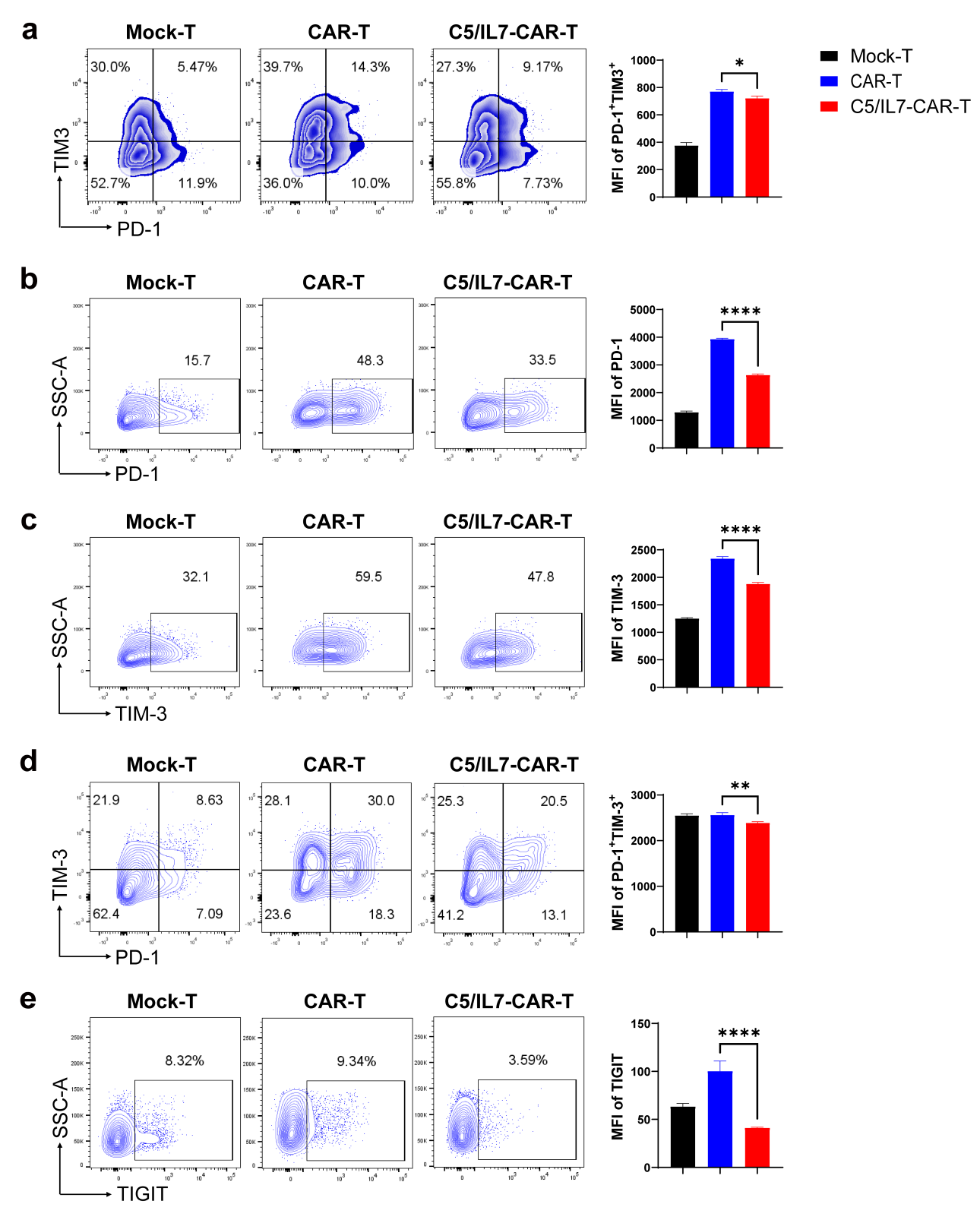


**Supplementary Figure 3**. C5/IL7 reduces CAR-T cell exhaustion. (a) PD-1 TIM3 double positive CAR-T cell analysis in the presence of U-2OS; Mock-T, CAR-T, and C5/IL7-CAR-T cells were co-cultured with U-2OS for 3 days at an Effector to Target (E: T) ratio of 3:1 in a 48-well plate. The cells were subsequently stained with FACS antibodies and analyzed by flow cytometry to identify double-positive cells expressing both PD-1 and TIM-3, indicative of T-cell exhaustion. (b-e) Flow cytometry analysis of IL-2 dependent exhaustion of CAR-T cells; Mock-T, CAR-T, and C5/IL7-CAR-T cells were cultured without target cells for 10 days. The expression of PD-1(b), TIM-3 (c), double-positive PD-1+TIM-3+ (d), and TIGIT (e) were analyzed by flow cytometry. The representative FACS profiles (right) and MFI bar graph (left) are shown. Experiments were performed independently at least 3 times. One-way ANOVA was used in Tukey’s multiple comparison test and data represent Mean ± SD. *P < 0.05, **P < 0.01, ****P < 0.0001.


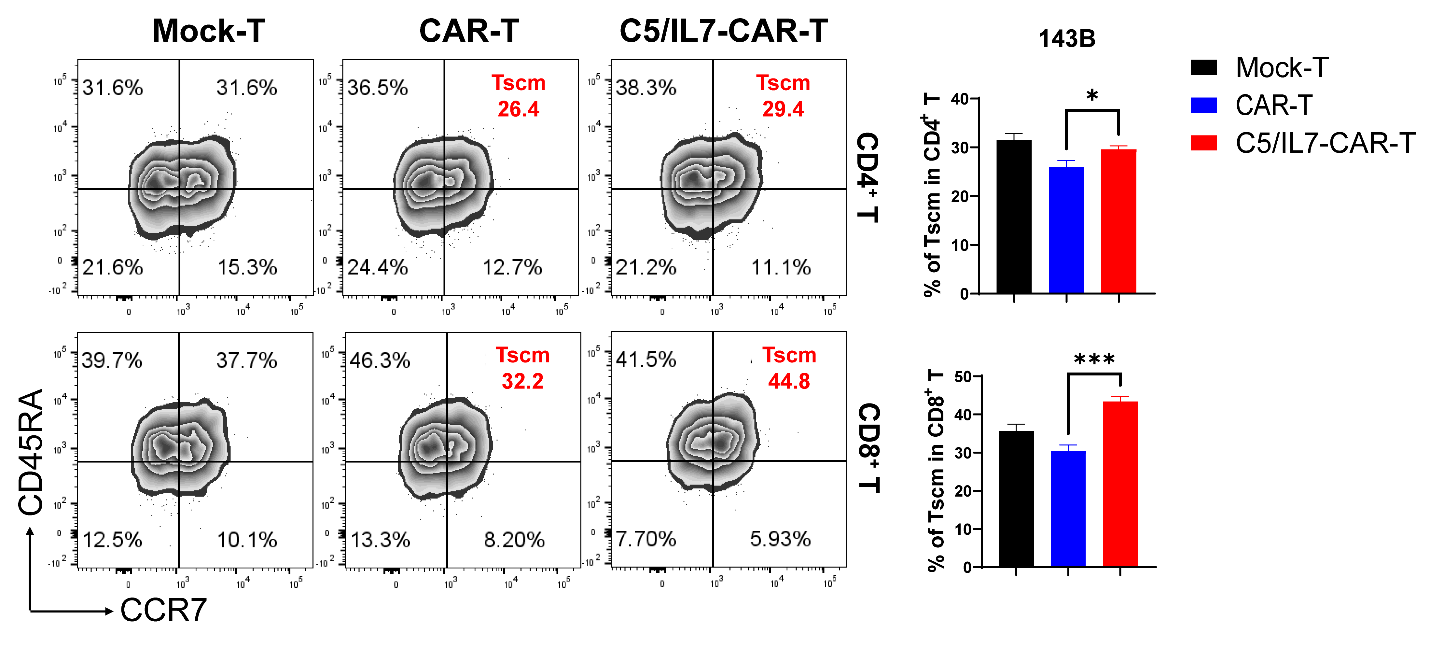


**Supplementary Figure 4.** C5/IL7-CAR exhibited a less differentiated T cell population against 143B cells. Cells from each experimental group after 5 days of co-cultured with 143B were analyzed by flow cytometry. Tscm: Stem cell memory T cell (CCR7+CD45RA+), Tcm: Central memory T cell (CCR7+CD45RA-), Tem: Effector memory T cell (CCR7-CD45RA-), Teff: Effector T cell (CCR7-CD45RA+). Experiments were performed independently at least 3 times. One-way ANOVA was used in Tukey’s multiple comparison test, and data represent Mean ± SD. *P < 0.05, ***P < 0.001.


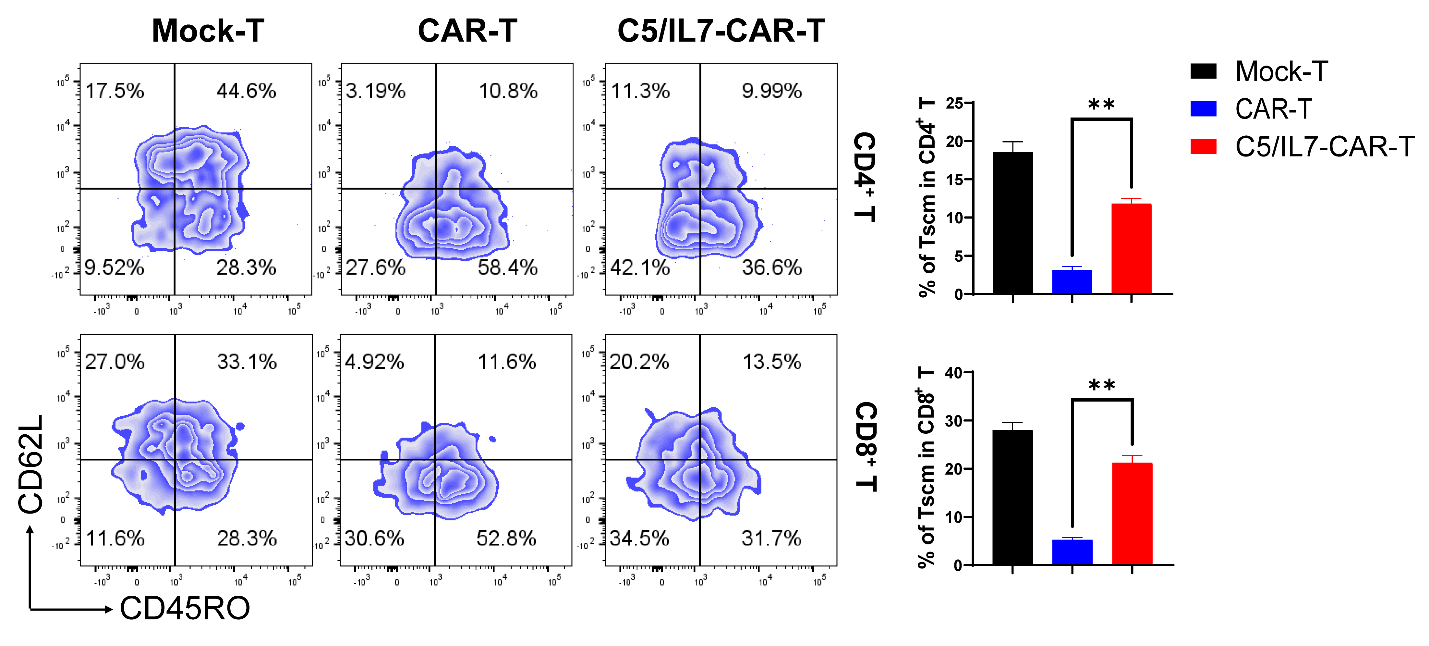


**Supplementary Figure 5.** C5/IL7-CAR exhibited a less differentiated T cell population against the U-2OS cell line. 3×105 Mock-T, CAR-T, and C5/IL7-CAR-T cells were cultured for 10 days, stained with FACS antibodies, and analyzed by flow cytometry. CD4+ T cell differentiation is depicted in the upper panel, and CD8+ T cell differentiation is illustrated in the lower panel. T cell subsets include Tscm: Stem cell memory T cell (CD62L+CD45RO-), Tcm: Central memory T cell (CD62L+CD45RO+), Tem: Effector memory T cell (CD62L-CD45RO+) and Teff: Effector T cell (CD62L-CD45RO-). Experiments were performed independently at least 3 times. One-way ANOVA was used in Tukey’s multiple comparison test, and data represent Mean ± SD. **P < 0.01.

**
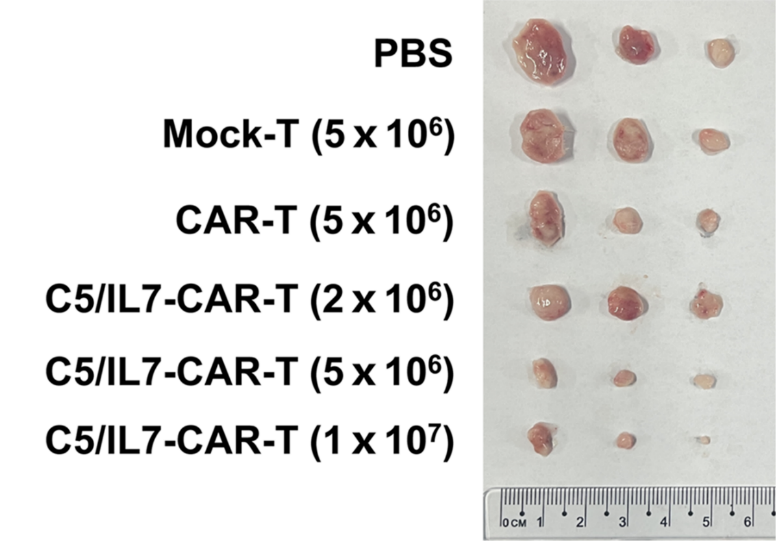
**

**Supplementary Figure 6.** Tumor size evaluation. Tumors were retrieved from the mice after treatment on day 27 (n=3) and subjected to digital imaging.

**
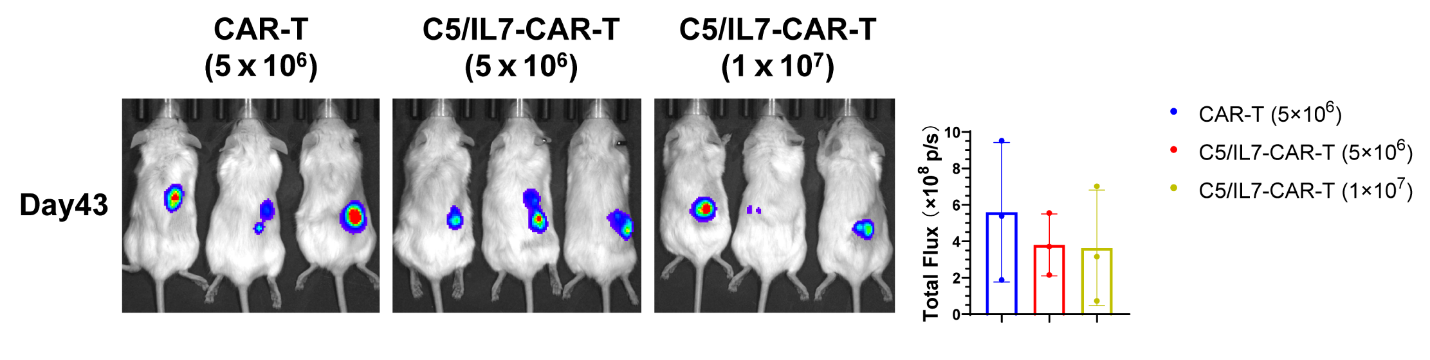
**

**Supplementary Figure 7.** Tumor bioluminescence imaging on day 43 post-treatment. The IVIS image depicts mice injected with 143B-luciferase cells, followed by treatment with CAR-T cells (left panel). The dot plot on the right indicates the total flux (photons per second), providing a quantitative measure of bioluminescence on day 43 after treatment.

**
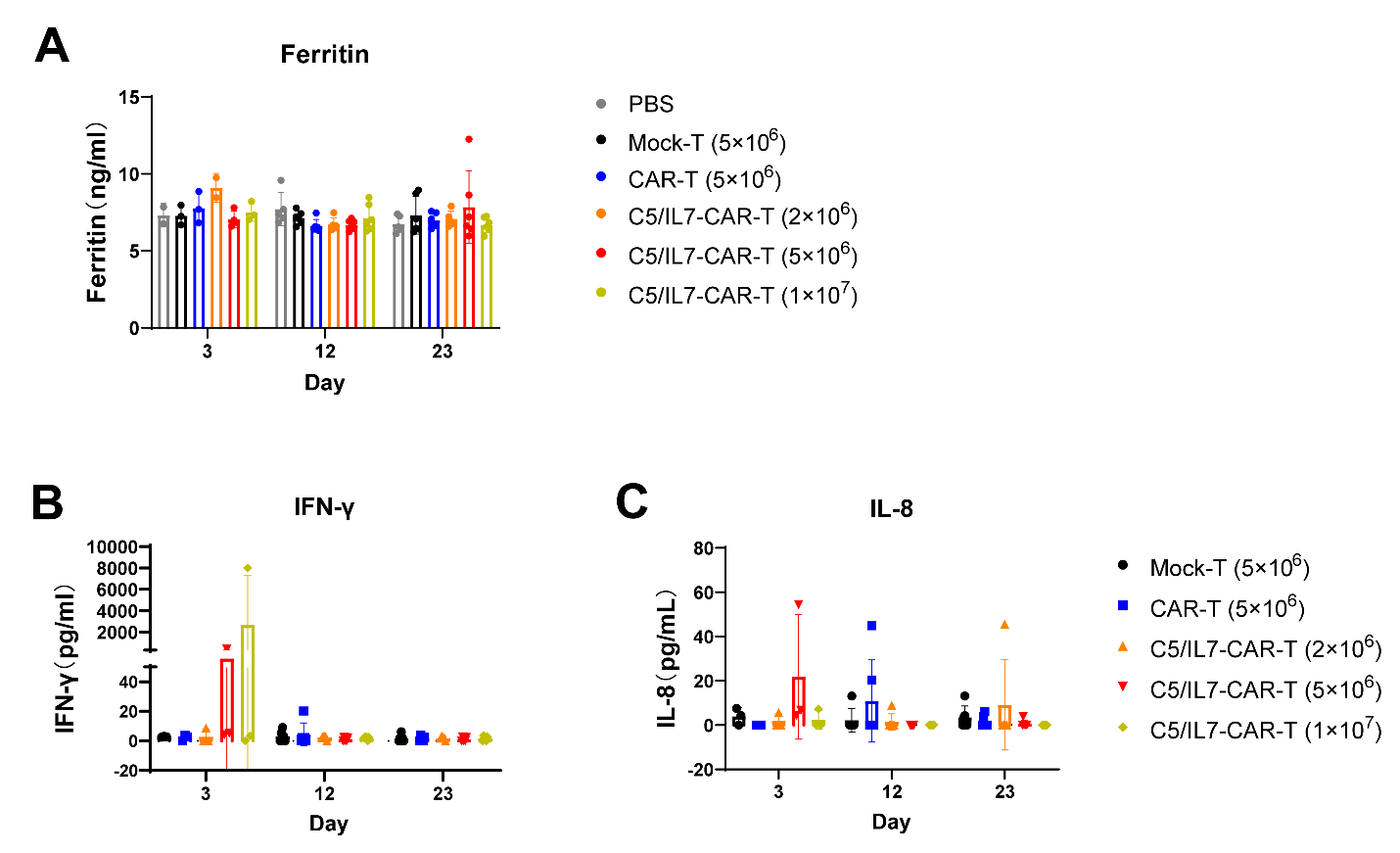
**

**Supplementary Figure 8.** Ferritin, IL-8, and IFN-γ levels in blood plasma. Peripheral blood was obtained from tumor-bearing mice on distinct days (days 3, 12, and 23) post-CAR-T treatment, centrifuged at 3000rpm, and the supernatant stored at -80°C. (A) Ferritin levels in each group was analyzed using ELISA. (B-C) Cytometry Bead Array (CBA) was employed for the analysis of IFN-γ and IL-8 expression to detect cytokine release syndrome (CRS), respectively.

**
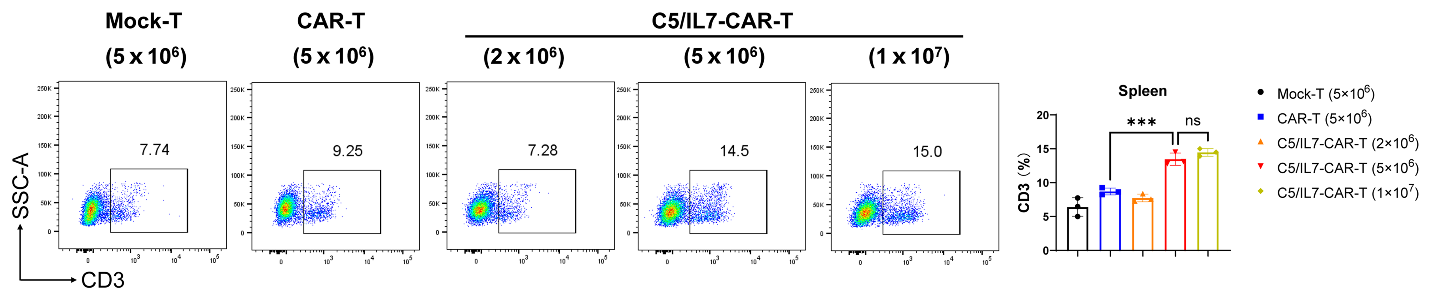
**

**Supplementary Figure 9.** T cell quantification in mice spleen after treatment. On day 27, mice (n=3) from each group were sacrificed, and spleen cells underwent single-cell suspension preparation. The CD3 population was measured via flow cytometry, using BV421 anti-human CD3 antibody staining for 30 minutes. CD3 cell percentage is depicted in FACS files on the left panel and in the column chart on the right side.

**
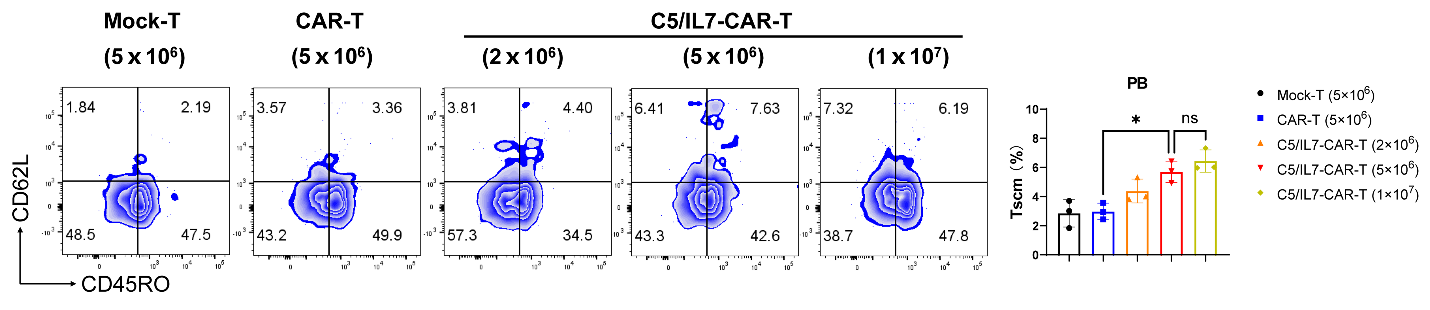
**

**Supplementary Figure 10.** T cell differentiation in peripheral blood. Flow cytometry analysis of Tscm (Stem cell memory T cell) proportion in peripheral blood of mice (n=3) from each experimental group on day 27 post-treatment. CD45RO- and CD62L+ cells were measured within CD3+ gated cells. Representative FACS files (left) and a corresponding column chart (right) depict Tscm distribution in each group.


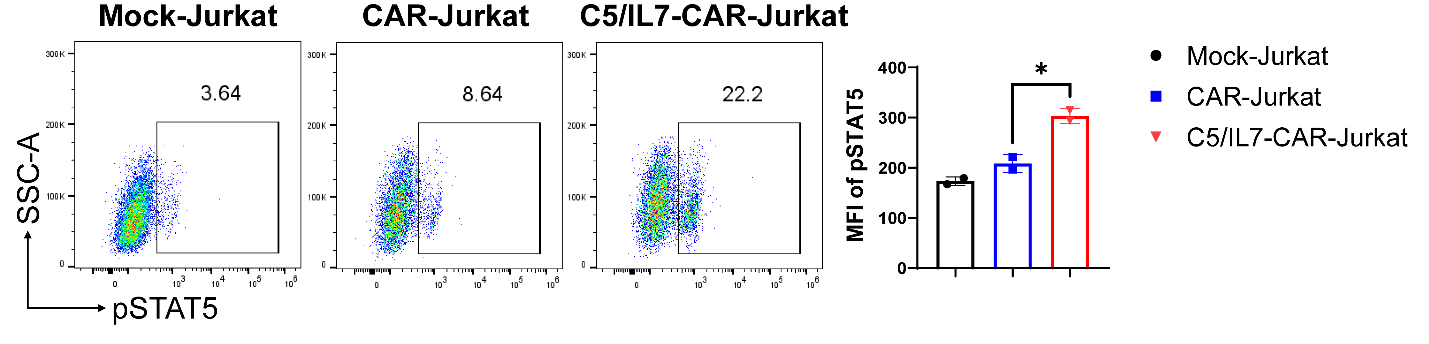


**Supplementary Figure 11.** pSTAT5 expression in Jurkat cells. Jurkat cells (1×106) were transduced with Mock, CAR, and C5/IL7-CAR lentivirus for 48 hours and subsequently co-cultured with U-2OS target cells at an E: T ratio of 3:1. After 16 hours, cells were collected and stained with PE anti-human STAT5 antibody, followed by flow cytometry analysis. Representative FACS files are presented on the left, while the MFI bar graph is on the right. Experiments were performed independently at least 3 times. One-way ANOVA was used in Tukey’s multiple comparison test, and data represent Mean ± SD. *P < 0.05.

**Supplemental table**

**Supplemental table 1:** List of antibodies used to perform Flow cytometry

| **Antibody Name** | **Company** | **Clone** | **Catalog number** |
| --- | --- | --- | --- |
| FITC Anti-human CD3 | Biolegend | OKT3 | 317305 |
| BV421 Anti-human CD3 | Biolegend | OKT3 | 317344 |
| APC Anti-human CD4 | BD Biosciences | RPA-T4 | 555349 |
| FITC Anti-human CD4 | BD Biosciences | RPA-T4 | 555346 |
| PE Anti-human CD8 | eBioscience™ | RPA-T8 | 12-0088-42 |
| PE/Cy7 Anti-human CD8 | eBioscience™ | SK1 | 25-0087-42 |
| APC Anti-human NKG2D | BD Biosciences | 1D11 | 558071 |
| BV421 Anti-human CXCR5 | BD Biosciences | RF8B2 | 562747 |
| PE Anti-human MICA/B | Biolegend | 6D4 | 320906 |
| APC Anti-human CD69 | Biolegend | FN50 | 310910 |
| PE/Cy7 Anti-human CD107a | Biolegend | H4A3 | 328618 |
| PE/Cy7 Anti-human GzmB | Biolegend | QA16A02 | 372214 |
| APC Anti-human IFN-γ | Biolegend | 4S.B3 | 502512 |
| PE Anti-human PD-1 | Biolegend | EH12.2H7 | 329906 |
| APC Anti-human TIM-3 | Biolegend | F38-2E2 | 345012 |
| BV421 Anti-human TIGIT | Biolegend | A15153G | 372709 |
| PE/Cy7 Anti-human TIGIT | Biolegend | A15153G | 372713 |
| PE Anti-human Bcl-2 | Biolegend | 100 | 658708 |
| APC Anti-human CD62L | Biolegend | OX-85 | 202916 |
| AF700 Anti-human CD62L | Biolegend | DREG-56 | 304820 |
| PE Anti-human CD45RO | BD Biosciences | UCHL1 | 555493 |
| PE/Cy7 Anti-human CD45RA | Biolegend | HI100 | 304125 |
| FITC Anti-human CCR7 | Biolegend | G043H7 | 353216 |
| APC Anti-human Ki-67 | Biolegend | Ki-67 | 350513 |
| PE Anti-human pSTAT5 | eBioscience™ | SRBCZX | 12-9010-42 |
| APC Annexin V | Biolegend | - | 640920 |
